# Trauma and the acute care surgery model – should it embrace or replace general surgery?

**DOI:** 10.1186/1757-7241-17-4

**Published:** 2009-02-04

**Authors:** Kjetil Søreide

**Affiliations:** 1 Department of Surgery, Stavanger University Hospital, Stavanger, Norway; 2 Department of Surgical Sciences, University of Bergen, Bergen, Norway

## Abstract

The specialties dealing with emergency medicine and emergency surgery are in need for a new roadmap. While the medical and surgical management of emergency conditions very often go hand-in-hand, issues relating to emergency and trauma surgery have particular concerns, which are global in magnitude. Obviously, choosing a career dealing (solely) with emergencies and trauma is associated with concerns related to lifestyle issues and, for surgeons, maintenance of adequate operative experience with the increased non-operative management. Also, dealing with patients' whose outcome may be dismal with high associated morbidity and mortality is often not viewed as rewarding. The global flux of medical students away from general surgical training and trauma surgery in particular is an example of how recruitment to specialties dealing with uncomfortable, unpredictable, and "out-of-office-hours" work may be in dire straits. How surgeons around the world will deal with this challenge will likely be diverse and tailored according to the needs of any given region, be it North America, Europe, or Scandinavia. However, refurnishing the training in General Surgery in order to ensure proper care for acute surgical illness and trauma appears mandated in order to keep in line with the centennial words of Halstead that *"every important hospital should have on its resident staff of surgeons at least one who is well and able to deal with any emergency that may arise*".

## Commentary

The specialties dealing with emergency medicine and emergency surgery is at a crossroads with the need for new roadmaps, finding new identity and redefining content [1-4]. While the medical and surgical management of emergency conditions very often go hand-in-hand, issues relating to emergency and trauma surgery have particular concerns, which are global in magnitude. Obviously, choosing a career dealing (solely) with emergencies and trauma is associated with concerns related to lifestyle issues and, for surgeons, maintenance of adequate operative experience, as well as dealing with patients' whose outcome may be dismal with high associated morbidity and mortality [5]. The global flux of medical students away from general surgical training and trauma surgery in particular is an example of how recruitment to specialties dealing with uncomfortable, unpredictable, and "out-of-office-hours" work may be in dire straits [6-10].

## 'General' vs 'one-organ' surgeons

**Figure 1:**
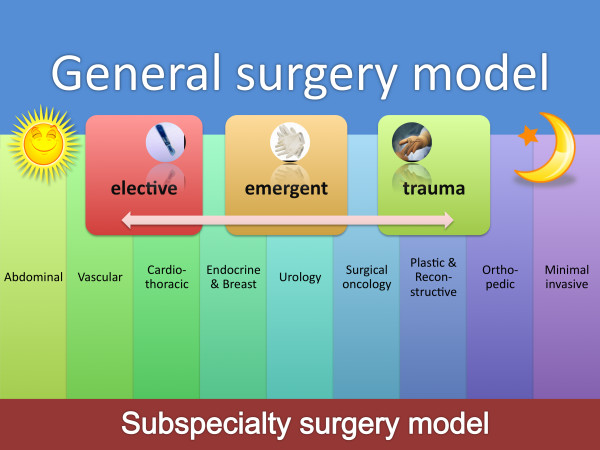
**Models for surgical care – specialized vs general**. The general surgery, day- and-night coverage of a wide range of elective to emergent surgical conditions (upper part of figure) is being replaced by expert surgeon (supra)-subspecialists hampering the surgical care of emergent conditions and trauma patients.

The current way surgeons deal with emergencies and trauma is very heterogeneous when viewed from an international perspective. Differences exist in focus of care, level of training, systems development and maturation worldwide – with each region having local variants and solutions to the problem [11-16]. For example, the best general description of the "European trauma care model" is that "there is none" – dislikes are more prominent than the likes in Europe [16,17]. In particular, trauma and emergency surgery has had no prominent place in most European countries, with no formal trauma surgical specialists in most countries. Except for trauma orthopedic surgeons of central Europe (the German "Unfallchirurg"), most trauma patients are cared for by general surgeons with/without some kind of subspecialty training level. Over the past decades this approach has changed, mostly due to an increased level of subspecialisation (figure 1). Fewer surgeons are being "general" by nature and delivering surgical care day for any given elective, emergent or trauma patient. Rather, surgeons are more often tending to "organ-specific" surgeon practices ("breast surgeon"; "pancreatic surgeon"; "colorectal surgeon") with no or only little competence in trauma and emergency surgical care. This has led a shift in focus from general surgery with broad spectrum of diseases and surgical techniques, to e.g. dedicated "abdominal surgeons", which again are in some centers focusing strictly on "upper gastrointestinal" or "lower"(= colorectal) surgery; and some again only on e.g. esophageal, or hepato-biliary pancreatic surgery. Often this has led to a shift away from, or simple exclusion of, emergency and trauma patient care. In some areas even colorectal surgeons would defer to "diseases of the appendix" as belonging to the "general surgeon", reflecting the narrow point of care.

**Figure 2:**
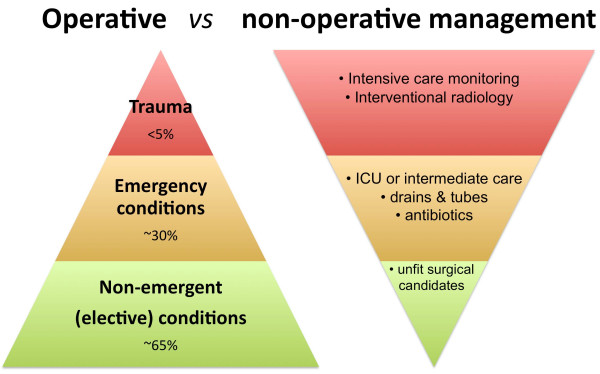
**Surgical exposure in elective, emergent and trauma conditions**. The majority of trauma patients are managed non-operatively, as influenced bu adjacent specialties such as interventional radiology. Including emergent conditions in the surgical armamentarium would ensure a wider range of surgical exposure and experience with critically ill patients, while including elective cases would ensure technical skills training and a better working lifestyle.

Consequently, the "omnipotent general surgeon" of the past who operated on all and everything is in principle now declared "dead". Further, with the dramatic decrease in operative volume for trauma ("good for patients – bad for surgeons"), the "trauma surgeon" of the past has become extinct. Trauma care is thus in need for resuscitation and revitalization. Further, not only trauma surgery has seen a dramatic increase in non-operative care. Controversies still exist with regard to the appropriate management of appendicitis [18], and even perforated diverticulitis with general peritonitis can be managed conservatively with laparoscopic lavage and no surgical resection [19]. As treatment paradigms shift and surgical emergency disease management evolve, we need properly trained surgeons that are willing in pursuing the optimal emergency care (surgical or non-operative) for specific conditions in selected patients (figure 2). As a consequence surgical training will have to change.

General surgical training in Norway no longer requires formal training in orthopedic surgery and, orthopedic surgeons no longer need to do general surgery in their rotations [6]. Breast and endocrine surgery is being separated from general surgery, Vascular surgery is increasingly being managed by minimal-invasive techniques, and increasingly often the gastrointestinal surgeons are being left for covering emergency surgery conditions still requiring emergent and urgent operative care. Overall, the general surgery workforce has followed a trend of increased specialization, with young surgeons gravitating toward specialties that are perceived to have a better lifestyle [15]. This development is seen worldwide and has led to troublesome gaps in the emergency surgery call schedule at many institutions.

## Moving from 'agenda' to 'curriculum' to 'patient care'

**Figure 3:**
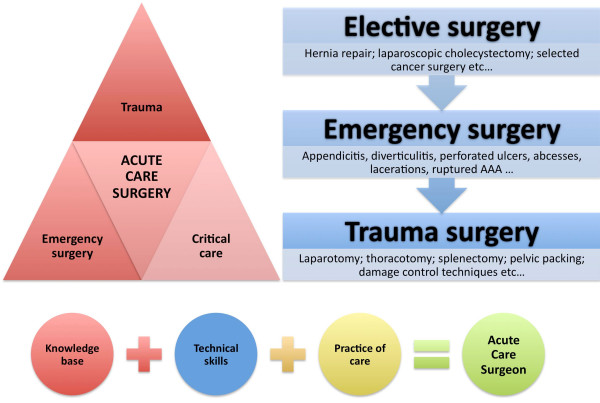
**The concept of Acute Care Surgery**. The knowledge base (upper left corner) and technical skills (upper right corner) should, together with the local requirements and range of practice (bottom part), address the scope of the Acute Care Surgeon.

Trauma and critical care surgeons in the USA have reexamined their role based on these concerns and the realization that surgeon resources for the injured patient are in jeopardy [20,21]. The new emphasis on non-trauma emergency surgery required an image change and thus a new name introduced as "Acute Care Surgery" [22]. After much work over the past several years, a model of "Acute Care Surgery" has emerged and a training curriculum has been proposed [22-25]. For US candidates, this concept is based on three major direction included into one (figure 3): trauma surgery, surgical critical care and emergency general surgery [26]. Some US centers already have begun assimilating acute care surgery into their departments with good results for their patients [27-30]. Others have combined elective, emergent and trauma surgery workload in their practice with positive benefits [31]. Increased operative volume in treating complex, but interesting patients is an immediate benefit to this approach with operative experience maintained by doing more elective and emergency surgery (figure 3).

Several challenges and opportunities associated with the implementation of this model have yet to be evaluated and overcome before a wider implementation – as of 2008 (American College of Surgeons Clinical Congress, San Francisco, October 2008) there are three institutions in the US with certified programs to educate Acute Care Surgeons. Many more are expected to follow in due course. However, concerns from general surgeons in rural and/or district hospitals in US have been echoed by colleagues in Scandinavia as well – will the general surgeon disappear and be replaced? And if yes, by whom? Or should the focus on trauma and emergency surgery in the ACS model embrace the general surgeon?

A definite answer has yet to appear, but from conference discussions and current reports, the Acute Care Surgery model is to be one of inclusion rather exclusion. Agreements appear to exist in that having only one model of Acute Care Surgery will be ineffective [14,20,32-36]. Several models must be created based on patients' needs and fitted to the local/regional logistical requirements of whether the surgeon practices in resource-rich or resource-limited environments (figure 3). Logistics and need should drive the skill set and assure proficiency, rather than turf or opinion. Designed with flexibility, Acute Care Surgery has the potential to have widespread application in urban, rural, remote, and military environments [20].

In the US, the programs for training acute care surgeons are recommended to be tailored to the appropriate care needed for that particular region and/or hospital – however minimum standards are set with the current curriculum [24,25,36]. As a consequence, orthopedic surgery may or may not be required as a central part of training. Likewise, neurosurgery skills may or may not be needed for the acute care surgeon, as some centers will have the available subspecialty expertise present. However, this is still heavily debated in the US [36-39].

Many practicing surgeons will say that they are already doing Acute Care Surgery – and by many means they probably are correct – e.g. appendicitis, perforated hollow viscus organs, ruptured aortic aneurysms, pancreatitis etc are in most cases dealt by 'general surgeons' often also caring for the (occasional) traumatized patient. What is lacking, however, is a structured training in trauma, emergency general surgery and intensive care among current general and subspecialty surgeons. Thus, rather than developing an entirely new specialty replacing the general surgeon, focus should be on an increased need for structured training, knowledge base and technical skills in surgical management of trauma and emergency conditions (figure 3).

## Embracing emergency skills in general surgical training

This has now been brought to the "sketch board" in refurnishing the training of Norwegian general surgeons, in order to include a curriculum that will, to a wider extent, address training issues of trauma and emergency surgery. The need to redirect training, with a "common trunk" of core surgical skills before subspecialization, will be important to attract future trainees to the field [1,26]. Further, the possibilities to obtain further subspecialty knowledge and skills in Acute Care Surgery within general surgery training will be important both for the rural/district hospitals in need for a surgeon who can deal with emergencies "24-7-365", as well as the academic trauma centers that see a great number of traumatized and/or critically ill surgical patients on a daily basis. This would be in line with the centennial words of William S. Halsted (1852–1922), that "*Every important hospital should have on its resident staff of surgeons at least one who is well and able to deal with any emergency that may arise*" [40]. To paraphrase a word on good patient care "the key to good [emergency] patient care, is to actually care for the [critically ill] patient". In doing so we need the surgical knowledge base and the operative skills to appropriately deal with trauma and emergencies. How surgeons around the world will deal with this challenge will likely be diverse and tailored according to the need of any region. Refurnishing the training in General Surgery may embrace the concepts built into the Acute Care Surgery model and ensure proper care for acute surgical illness and trauma – else the general surgeon, as viewed in the past, may be replaced and become extinct.

## Competing interests

The author declares that they have no competing interests.

## Authors' contributions

KS perceived the concept and drafted the article.
